# Not That Kind of a God

**Published:** 1889-08

**Authors:** M. J. Savage


### NOT THAT KIND OF A GOD.

" Were there no criticism to tell us that the Bible is not infallible, to tell us of the natural origin of all religions ; were there no criticism to tell us of the natural origin of creeds ; were there no science to tell us that the old conception of the universe was as a baby's playhouse compared to the infinite majesty of what we now know to be true, to tell us that man has been on this planet hundreds of thousands of years ; had it not been demonstrated that man has been developed from lower forms of life,—were these things all unknown, the growing civilization of the world, the goodness of the human heart, would have made it impossible for the world any longer to believe in the cruel egotist sitting on the throne of the universe, and governing all merely for his own glory. The world is too good for that kind of a God any longer.

So you find that the churches of every name, though they claim to hold the creeds, do put on one side more and more those things that the reverence, and tenderness, and sympathy, and love, and goodness of the human heart will no longer bear. And so we hear men like Whittier saying, 'Dear friends, my human hands are weak to hold your iron creeds.' The revolt of the heart demands at last that the infinite God of the universe should be as good as a good man. These are the reasons why there is a break-up of the old orthodoxy ; why men do not any longer believe in and accept it.

And what is the significance of these reasons ? Does it mean that the world is less religious, less moral, less reverent ? Does it mean degeneracy, decay ? It means that this human race of ours, starting as a child, is on the road towards manhood ; that it is growing, that it has grown too intelligent, too tender hearted, too good, any longer to bear the intellectual contradictions, and puerilities, and crudenesses, and cruelties of the old theories of religion. We shall find, I believe, that the world has not outgrown religion, not even outgrown the church or the church idea, but that all we love, all we care for, not only remains, but is to go on, becoming ever more and more."—REV. M. J. SAVAGE.

